# A Multimodal Sensory Apparatus for Robotic Prosthetic Feet Combining Optoelectronic Pressure Transducers and IMU

**DOI:** 10.3390/s22051731

**Published:** 2022-02-23

**Authors:** Tommaso Fiumalbi, Elena Martini, Vito Papapicco, Filippo Dell’Agnello, Alessandro Mazzarini, Andrea Baldoni, Emanuele Gruppioni, Simona Crea, Nicola Vitiello

**Affiliations:** 1 The BioRobotics Institute, Scuola Superiore Sant’Anna, 56127 Pisa, Italy; elemarti.nove@gmail.com (E.M.); papapicco.vito@gmail.com (V.P.); filippo.dellagnello@santannapisa.it (F.D.); alessandro.mazzarini@santannapisa.it (A.M.); andrea.baldoni@santannapisa.it (A.B.); simona.crea@santannapisa.it (S.C.); nicola.vitiello@santannapisa.it (N.V.); 2 Department of Excellence in Robotics & AI, Scuola Superiore Sant’Anna, 56127 Pisa, Italy; 3 Centro Protesi INAIL, Istituto Nazionale per l’Assicurazione Contro gli Infortuni sul Lavoro, 40054 Bologna, Italy; e.gruppioni@inail.it; 4 IRCCS Fondazione Don Carlo Gnocchi, 20162 Milan, Italy

**Keywords:** prosthetics, wearable sensors, control, gait segmentation, optoelectronic sensors, inertial measurement unit

## Abstract

Timely and reliable identification of control phases is functional to the control of a powered robotic lower-limb prosthesis. This study presents a commercial energy-store-and-release foot prosthesis instrumented with a multimodal sensory system comprising optoelectronic pressure sensors (PS) and IMU. The performance was verified with eight healthy participants, comparing signals processed by two different algorithms, based on PS and IMU, respectively, for real-time detection of heel strike (HS) and toe-off (TO) events and an estimate of relevant biomechanical variables such as vertical ground reaction force (vGRF) and center of pressure along the sagittal axis (CoPy). The performance of both algorithms was benchmarked against a force platform and a marker-based stereophotogrammetric motion capture system. HS and TO were estimated with a time error lower than 0.100 s for both the algorithms, sufficient for the control of a lower-limb robotic prosthesis. Finally, the CoPy computed from the PS showed a Pearson correlation coefficient of 0.97 (0.02) with the same variable computed through the force platform.

## 1. Introduction

Lower-limb amputation affects millions of people worldwide. In the USA, for example, 185,000 people suffer limb loss each year, and these numbers are expected to double in the next 30 years [1]. The daily living activities can be affected by limitations in locomotion tasks in terms of performance and asymmetry of the gait pattern [2]. In particular, it has been estimated that both transfemoral and transtibial amputees increase their oxygen consumption during locomotion by over 25%, which is directly related to an increase in metabolic expenditure [3].

In recent years, lower-limb prostheses have remarkably advanced, as companies and academic institutions are increasingly investing resources in their development to improve the living standards of amputees. Generally, lower-limb prostheses can be divided into three main categories: passive, semi-active, and fully-active prostheses [4]. Passive energy-storage-and-return (ESAR) prostheses have become very popular as they can restore a more natural ambulatory pattern than non-ESAR counterparts [5]. Nevertheless, passive feet cannot provide net positive energy to the gait cycle. Semi-active prostheses can vary their inner mechanical properties (e.g., resistance, mainly by using dampers) or elastic properties (through smart clutches) to adapt to different terrains or locomotion phases and tasks [6]. However, they are usually unable to provide net positive energy to amputees during stance, ultimately limiting the potential quality of the gait biomechanics restored by the prosthesis [2]. Such limitations have motivated researchers and companies to investigate active, powered/robotic solutions for lower-limb prostheses. Notably, the efficacy and the acceptability of such devices heavily rely on the implementation of non-invasive sensory systems that could allow the autonomous and reliable functioning of prostheses. Indeed, the reduction of the energetic cost of walking strongly depends on the ability to detect relevant gait events and provide net positive power with precise timing [7,8,9]. In this scenario, the sensory system of a prosthetic leg should be able to infer the user’s motion (e.g., through the interaction with the environment) to input intelligent, adaptive algorithms and permit a consistent and responsive actuation of the prosthesis [10,11,12].

Encoders, load cells, inertial measurement units (IMUs), and plantar force-sensing technologies—either alone or in combination—are commonly used to detect the most relevant gait events in lower-limb wearable robots, including robotic prostheses [7,13,14,15]. In particular, sensory fusion from multiple inputs proved to increase the richness and the redundancy of information, maximizing the accuracy and the reliability of the segmentation algorithms, although often at the price of increased mechatronic complexity and computational costs, and less practical wearability [16,17].

For instance, load cells have been widely used in robotic prostheses to measure the torque at the joint level and to detect contact with the ground [7]. However, they have certain limitations: the cost, which generally increases with the amount of information provided and, therefore, the number of axes they integrate; and the safety, as they are not a redundant sensory system and in case of malfunction, they can have a detrimental impact on the gait segmentation algorithm and the overall system stability, with risks of injury for the end-users [18,19].

To simplify the structure integrating the load cell, uniaxial force or moment load cells have been combined with additional sensors to reach the information needed to control active prostheses. For example, in a study of Sup et al. [20,21], an anthropomorphic transfemoral prosthesis implemented a 3-axial off-the-shelf load cell and strain gauges under the ball and the heel, and the latter was used to detect gait events. In Hu et al. [22], the authors instrumented an insole with two load cells, and they used a model-based algorithm to detect stance and swing phases in real-time (RT). An alternative approach to contain weight and encumbrance is represented by a custom force/torque sensor housed within a pyramid adapter, as presented by Gabert et al. [18].

Other solutions include insoles [23,24] or spare pressure sensors connected at the foot-shoe interface [25]. The F-scan measurement system (Tekscan Inc., South Boston, MA, USA) has been implemented in various applications [26]. At the same time, other prototypical technologies have included optoelectronic sensors [27,28] and force-sensitive resistors (FSR) [29]. IMUs—alone or in combination with pressure sensors—have also been applied to complement pressure sensory information during the swing phase [30,31]. When compared to load cells, pressure sensors and IMUs are generally cheaper but less accurate and reliable, with pressure sensors often also characterized by poor durability [24]. Though these characteristics are seldom benchmarked, they constitute a practical barrier for adopting the sensors in real-case applications.

Hence, while the state of the art research highlights that the use of multi-modal sensory information combining pressure sensors and IMUs can serve several control purposes (including gait-phase estimation [32], decoding of users’ movement intention [16], terrain recognition [33], and provision of augmented sensory feedback [28,34]), the integration of pressure sensors into the prosthesis structure/mechatronics has not been thoroughly addressed; in particular, the majority of studies use pressure-sensors outside the cosmetic cover, while there are only a few examples of prostheses that integrate pressure sensors at the interface between the ESAR foot and the cosmetic cover [25].

This work targets the development and in-lab experimentation of a multi-modal sensory apparatus for detecting gait events (i.e., heel strike and toe-off) relevant for the control of active prostheses based on ESAR feet. The proposed solution incorporates two sensing technologies into a commercial carbon-fiber prosthetic foot. One technology is a matrix of optoelectronic pressure-sensitive transducers [27,28] placed in between the carbon lamina and a custom cosmetic foot cover, and the second is a commercial IMU on the tibial part of the carbon foot. Such an integrated solution has the advantage that the end-users can easily wear the prosthesis within the shoes without the need to place any additional sensors (e.g., sensorized insoles). Due to the sensor technology, the proposed solution offers two additional advantages: first, the pressure technology provides quali-quantitative information on the vertical ground reaction force (vGRF) and the progression of the center of pressure (CoP) along the antero-posterior direction of the foot, and second, the matrices of the sensors can be easily re-arranged to fit different off-the-shelf ESAR feet.

## 2. Materials and Methods

The following sections are devoted to illustrating the requirements, the design, and the in-lab experimentation of the sensory apparatus for a prosthetic foot; attention is paid to the description of the transducers’ working principle and arrangement, as well as to the electronic components, the structural parts, and the algorithms for the gait segmentation. Hence, the technology has been designed at the Wearable Robotic Laboratory of The Biorobotic Institute (Scuola Superiore Sant’Anna, Pisa, Italy), and it has been implemented within a prosthetic foot that forms the structural part of a robotic TransTibial Prosthesis (WRL TTP) within the framework of the MOTU (G.A. n. PPR-AI 1-2) and MOTU++ (G.A. n. PR19-PAI-P2) projects promoted by Centro Protesi INAIL (Vigorso di Budrio, Italy). The working principle and mechatronics design of the WRL TTP is out of the scope of this work; however, in analogy with other robotic prostheses, the control of the WRL TTP, which has been designed to inject energy during the push-off phase of the stride, requires timely detection of the different gait phases. Therefore, the experimentation of the sensory system aimed at assessing its capability to promptly detect relevant gait events (e.g., heel strike and toe-off).

### 2.1. Concept Description

The design of the sensory apparatus, which we name from now on the Sensorized Prosthetic Foot (SPF), followed predetermined functional and technical specifications, as well as practical feasibility and usability constraints. The system’s design considered requirements about size, weight, and geometrical factors to properly integrate the sensory apparatus into a commercial prosthetic foot, besides accuracy and precision [24,35]. Overall, the design of the sensory system of the prosthetic foot followed three main functional goals.

**Timely and reliable detection of gait events relevant for the control of a robotic lower-limb prosthesis.** The primary objective was to develop a sensory system that could detect the two main phases of the gait cycle, functional to the control of a robotic prosthesis, i.e., stance and swing, with a maximum time error of 0.100 s [30,36,37].

Reliability was addressed by fostering redundancy. Indeed, the sensory system integrates two sources of data functional to the gait segmentation, namely: (i) an IMU provides linear acceleration and rotational speeds of the foot, and (ii) the pressure sensors provide an estimate of the vertical component of the foot-ground interaction force, i.e., vertical ground reaction force (vGRF), as well as its instantaneous point of application along the foot longitudinal axis, i.e., y-coordinate of the center of pressure (CoPy).

Segmenting the gait cycle in the above-mentioned two phases requires the identification of the following gait events: the heel-strike (HS) and the toe-off (TO).

**Low risk of entanglement/collision with the external world**. To promote the use of lower-limb robotic prostheses in scenarios of daily life, the sensory system was designed to avoid any possible collision of its constituents with the external environment. Thus, it has to be self-contained within a custom foot cover, with mechanical properties (i.e., stiffness/strength) comparable to one of the off-the-shelf solutions [38,39]. Besides, the sensory system was designed to be lightweight and compact [36].

**Compliance with most off-the-shelf prosthetic feet.** The sensory system was designed to be easily housed into energy storage and release (ESAR) feet (e.g., Pro-Flex^®^, Vari-Flex^®^, Össur, Reykjavik, Iceland), and therefore, need to be compatible/adaptable to the size and shape of several prosthetic feet. This level of versatility is mainly provided by three features of the proposed design: (i) number and location of the pressure sensors under the prosthetic foot sole can be adapted (e.g., by locating a variable number of sensors under the forefoot or the heel) depending on the specific layout of the target prosthetic foot, (ii) the electronics are housed in a box that can be conveniently attached to any prosthetic foot frame, and (iii) the routing of all electrical wires was conceived to be fully contained inside the foot cover.

### 2.2. Mechatronics

**Figure 1:**
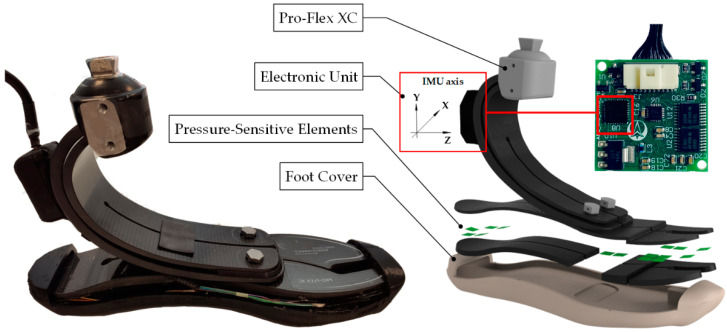
Overview of the system with an exploded view of the SPF: the electronic unit is enclosed inside a black plastic box, and it is endowed with an IMU (axis orientation is reported); the prosthetic foot, i.e., Pro-Flex XC, Ossur^®^; pressure-sensitive elements are made up of black silicone pads and PCBs; a 3D printed foot cover encloses the sensing elements and cables to prevent failures.

The SPF consists of the following modules: (i) a commercial prosthetic foot (i.e., Pro-Flex XC, Ossur^®^); (ii) a set of pressure-sensitive elements, called tactels; (iii) the foot cover; and (iv) an electronic unit (embedding the IMU) for data collection and communication with other devices, such as the main control unit of an active/robotic prosthesis (Figure 1).

#### 2.2.1. Pressure-Sensitive Transducers

Vertical ground reaction force and the y-coordinate of the center of pressure are estimated through a matrix of optoelectronic pressure-sensitive transducers located under the prosthetic foot.

**Figure 2:**
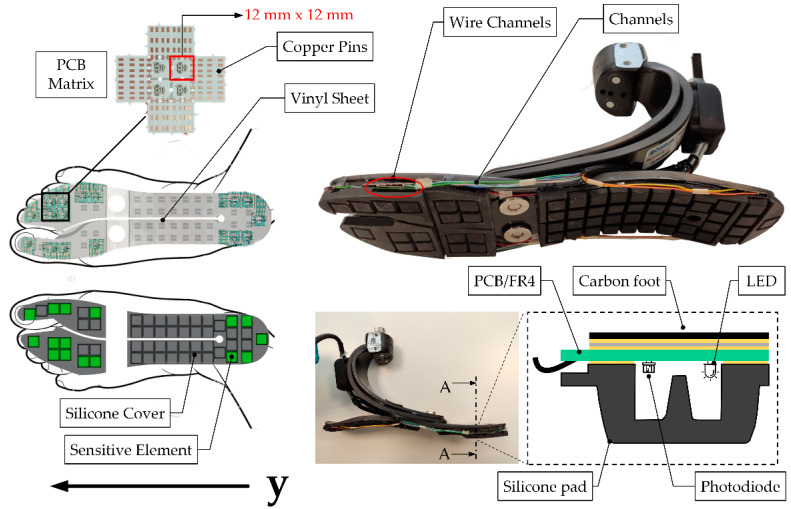
Positioning of sensitive elements below the carbon foot; highlighted are the modularity of PCBs, the sensitive elements in green, wiring paths, and the final assembling of the SPF. The vinyl sheet is used for assembling PCBs in a pre-defined configuration. The main components of the tactels are reported in section A-A: the silicone pad, the photodiode, the LED, and PCB.

The pressure-sensitive elements are based on a patented technology initially designed to measure the force at the physical human-robot interface in exoskeletons and then translated to plantar pressure applications with in-shoe foot soles [27,28]. Each sensitive element (tactel) consists of an LED (Kingbright GmbH, Taipei, Taiwan) and a photodiode (Avago Technologies, San Jose, CA, USA) welded on a printed circuit board (PCB, four layers with an overall thickness of 0.4 mm). The PCBs are coupled to a silicone pad whose section has the shape of an empty pyramidal frustum with a squared basis (12 × 12 × 6 mm^3^, see Figure 2). The top part of the silicone pad has an internal curtain in the middle; a force applied onto the upper basis of the pyramidal frustum deforms the silicone bulk, lowering the curtain, which proportionally obstructs the passage of light from the LED to the photodiode, therefore causing a change in the photodiode output voltage $V_{i}$. The voltage is therefore related to the vertical force $F_{i}$ acting on the ith pressure transducer. The material and the geometry of the single element determines a 4th-grade polynomial relationship between the applied force and output voltage, as reported in [28]:

(1) $$F_{i}=p_{1}V_{i}{}^{4}+p_{2}V_{i}{}^{3}+p_{3}V_{i}{}^{2}+p_{4}V_{i}+p_{5}\;(N),$$

where $p_{1}=186.1$, $p_{2}=224.5$, $p_{3}=64.76$, $p_{4}=-18.59,$ and $p_{5}=0$.

The characterization function represents the relationship between a quasi-static normal force applied on the upper base of the sensor and the output voltage [27,28,40].

As shown in Figure 2, the silicone sole has several pads for the accommodations of PCBs, though not all of them can sense the pressure since not all of them are instrumented with optoelectronic components. Indeed, due to the specific layout of the prosthetic foot, the designer could decide the number and which silicone pads shall be instrumented; clearly, the higher the number of instrumented pads, the higher the overall system complexity. Figure 2 shows the case where 16 tactels were instrumented: 5 under the heel and 11 under the forefoot. This layout was found to be a reasonable trade-off between complexity and accuracy for the target off-the-shelf prosthetic foot. Once the layout of the sensing elements is defined, the silicone pads and the PCBs are bounded to the ESAR foot through a vinyl sheet and a layer of epoxy resin (RS Components Ltd., Corby, Northants, UK).

Given the layout of the sensing elements, it is possible to estimate the vGRF and the CoPy as follows:

(2) $$vGRF=\sum_{i=1}^{16}F_{i}\;,\;where\;F_{i}=\{\begin{array}{c} f\left(V_{i}\right)\;\;V_{i}\leq V_{T}\\ 0\;\;\;\;\;V_{i}>V_{T} \end{array}\;(N),$$

(3) $$CoPy=\{\begin{array}{c} \frac{\sum_{i=1}^{16}\left(F_{i}\cdot w_{y_{i}}\cdot y_{i}\right)}{\sum_{i=1}^{16}\left(F_{i}\cdot w_{y_{i}}\right)}\;\;\;\;\;vGRF\leq vGRF_{T}\\ NaN\;\;\;\;\;\;\;\;\;\;\;\;\;\;vGRF>vGRF_{T} \end{array}\;(\mathrm{cm}).$$

In an analogy to the methodology presented in [28], the contribution of each sensor is considered only when its output is below the threshold $V_{T}$, to minimize the impact of noise on the estimate of the overall vGRF (note: when pressed, each tactel outputs a negative voltage). CoPy is obtained by weighting the force contribution of each tactel ($w_{yi}$) by its distance from the heel. The geometrical coordinates of each tactel along the longitudinal axis of the foot, referred as $y_{i}$, span between 0 cm and 22.8 cm (Figure 2).

The SPF was designed to bear the weight of an amputee having a maximum body mass of 90 kg walking at 3 km/h [41]. The overall assembly increased the weight and foot-plate thickness of the ESAR prosthesis by 150 g and 0.55 cm, respectively.

#### 2.2.2. Foot Cover

**Figure 3:**
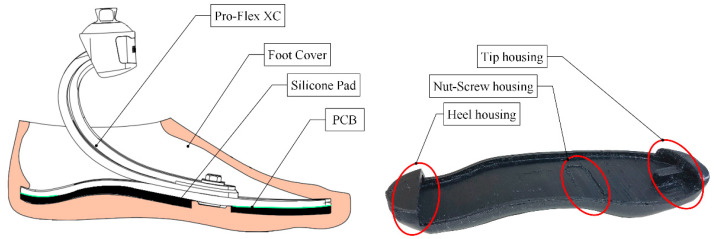
The commercial prosthetic foot, i.e., Pro-Flex XC, and the commercial foot cover (pink) are shown on the left of the picture. On the right, the components of the 3D printed foot cover that enable the prosthetic foot’s accommodation within the cover.

In this study, the passive prosthetic foot Pro-Flex XC (Ossür^®^, Reykjavik, Iceland) was selected, which is usually paired with an off-the-shelf foot cover foot in plastic foam [42]. This cover not only serves for cosmetic purposes but also has an essential mechanical function since its inner geometry guarantees a precise fit of the prosthetic foot using heel and tip housing (Figure 3). To comply with the space and the height added by the insertion of the pressure sensors, the foot cover was re-designed.

The design procedure of the foot cover involved several actions: (i) the sole geometry of the carbon foot was acquired via a portable 3D scanner Go! Scan20 (Creaform, Ametek Srl, Milan, Italy); (ii) the output files were post-processed using the software VXModel provided by the scanner manufacturer (Creaform, Ametek Srl, Milan, Italy) to clean the model from excess captured meshes; (iii) the final shape of the foot cover was designed through the help of the 3D CAD software Creo 2.0 (PTC, Boston, MA, USA). Finally, the foot cover was fabricated through a 3D printer (Delta WASP 2040, WASP Srl, Milan, Italy) implementing the fused filament fabrication (FFF) technology with FiloAlfa^®^ FiloFlex, a flexible filament of thermoplastic polyurethane (TPU, 55 Shore D, Gent, 1958). The resulting weight of the redesigned foot cover was 115 g.

#### 2.2.3. Electronic Unit

The electronic unit features a small plastic box housing a custom PCB (namely the data acquisition board, DAB) that reads the sensors’ signals. It is interfaced with the main control unit (the prosthesis control unit, PCU). The DAB acquires all the 16 analog signals output by the tactels and the signals of the 9-axis IMU embedded in the PCB of the DAB. Notably, the DAB is equipped with:

1. A precision 12-bit analog-to-digital converter (ADC, ADS7953, Texas Instruments, Dallas, TX, USA) that integrates an analog front-end including (i) an embedded multiplexer with 16 channels and (ii) an external operational amplifier (LMP7701, Texas Instruments, Dallas, TX, USA) configured as a non-inverting buffer for impedance adaptation and loading effect minimization;
2. A power management unit that generates a stable power source for the sensors and the circuitry of the DAB itself;
3. A 9-axis inertial measurement unit (IMU) MPU9250 (TDK/InveSense, Inc., San Jose, CA, USA);
4. Differential RS485 line drivers (AM26C31/32 Texas Instruments, Dallas, TX, USA) for reliable digital serial peripheral interface (SPI) communication with the main control unit of the WRL TTP through a wired line.

**Figure 4:**
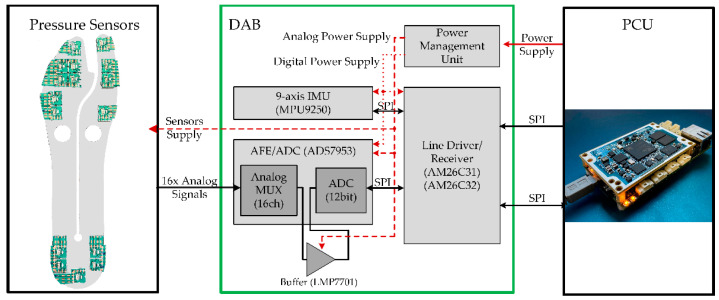
From left to right: pressure sensors arrangement below the prosthetic foot; Data Acquisition Board (DAB) architecture; Prosthesis Control Unit (PCU) for signal processing. The scheme of DAB comprises the IMU, the analog front end/ADC, the power management unit, and the differential line drivers.

The DAB is interfaced with the PCU via two differential SPI buses to enable the RT collection of all sensors signals (Figure 4). Differential SPI buses permit setting/reading the ADC and the IMU, respectively. The PCU is configured as a master in the SPI communication, and it performs the RT sampling of all sensors with a frequency of 1 kHz. For this study, the DAB was located on the rear part of the C-shaped structure of the ESAR prosthetic foot.

### 2.3. Algorithm for Gait Events Detection

To verify the performance of the developed technology concerning the timely detection of HS and TO, two different algorithms were developed relying on pressure and IMU signals, respectively. In this paper, the two algorithms are tested separately. Combining the output of the pressure sensors and IMU to enhance robustness is out of the scope of this work and will be addressed in future investigations.

The detection of the gait events based on the pressure sensors (“PS-based” algorithm) relies on a threshold for the vGRF, namely $vGRF_{T}$. When the estimated vGRF is above or equal to the threshold value, the foot is in contact with the ground (stance phase); alternatively, the foot is flying (swing phase). During the gait, the transition from swing to stance identifies the HS; vice versa, the transition from stance to swing identifies the TO. The minimum vGRF to detect TO and HS events was tuned within the range [10, 20] N.

**Figure 5:**
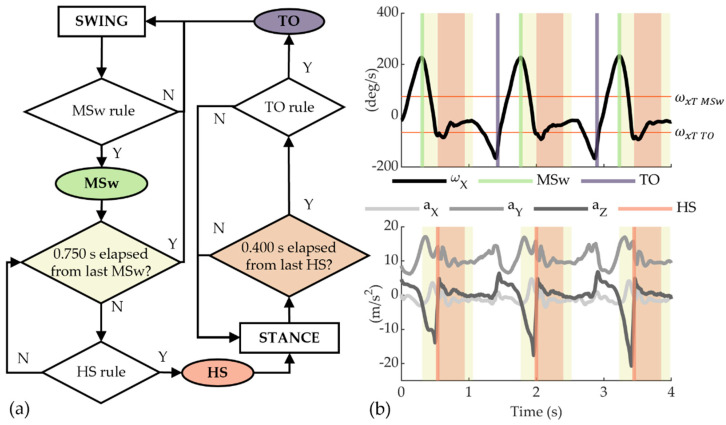
(**a**) Block diagram of the IMU-based algorithm for gait events detection; (**b**) Real-time gait events detection for an illustrative case. Vertical lines represent mid-swing (MSw), heel-strike (HS), and toe-off (TO) events. The yellow shaded area represents the observation window of 0.750 s after MSw, while the orange shaded one shows the idle period of 0.400 s after HS.

For the IMU signals, we considered a rule-based, finite-state machine algorithm (Figure 5), taking the following variables as input: the accelerations along the three axes, namely $a_{x}$, $a_{y},$ and $a_{z}$ (m/s^2^), and the angular velocity onto the sagittal plane, i.e., around the IMU *x*-axis, namely $\omega_{x}$ (deg/s) (“IMU-based” algorithm). At power on, the algorithm looks for a local maximum of $\omega_{x},$ which is higher than a pre-defined threshold $\omega_{xT\;MSw}$. The identification of this local maximum at the mid-swing (MSw) activates the search of the HS, which is identified as the time instant when both the following two conditions apply: (i) $a_{z}$ has reached a local maximum, and (ii) in the 30-millisecond time window before the occurrence of the local maximum of $a_{z}$, at least one of the three accelerations signals ($a_{x}$, $a_{y},$ and $a_{z}$) spanned a peak-to-peak range higher than a pre-defined threshold ($a_{T}$). In the case that no HS event is detected after 0.75 s from the detection of the MSw, then the search of the HS is stopped, and the algorithm waits for the occurrence of a new MSw event.

If an HS is detected, an idle period of 0.4 s elapses before the search of the TO starts. The TO event is found in the correspondence of a local minimum of $\omega_{x}$ lower than a pre-defined threshold $\omega_{xT\;TO}$. For the IMU-based algorithm, the following constant values were selected as thresholds: $\omega_{xT\;MSw}=75$ deg/s; $\omega_{xT\;TO}=-65$ deg/s; $a_{T}=7$ m/s^2^. All parameters considered in the analysis are listed in Table 1.

### 2.4. In-Lab Experimentation

In the experimentation of SPF, we compared its performance with a commercial force platform, which is commonly adopted in biomechanics/clinical gait studies [26,28,43]. The SPF foot was tested with eight healthy volunteers (sex: 6 males, 2 females; age: 29.4 ± 2.4 years; BMI: 19.2 ± 1.4 kg/m^2^). The inclusion criteria for the enrolment included age between 18 and 60 years, good health, and anthropometry that was compatible with the prosthetic foot and the custom adapters. The presence of cognitive disorders or physical conditions that could alter the physiological biomechanics (e.g., joint or articular pain, recent sprains or injuries, etc.) determined the exclusion of the candidate from the study. The study was conducted in accordance with the Declaration of Helsinki, and the protocol was approved by the local Ethics Committee of Scuola Superiore Sant’Anna (SSSA, Pisa, Italy; approval n° 6/2020; date of approval 22/05/2020). All subjects gave their informed consent for inclusion before they participated in the study. All experiments were carried out in the gait laboratory at the premises of the IRCSS Fondazione Don Gnocchi (Firenze, Italy).

Two reflective markers were placed on the heel and tip of the prosthetic foot, and their trajectories were recorded by a motion capture system (Smart-DX, BTS Bioengineering, Quincy, MA, USA). We used an external analog trigger to synchronize motion tracking data with the data acquired from the prosthetic foot. Moreover, markers data were analyzed to compute the velocity of the participants.

**Figure 6:**
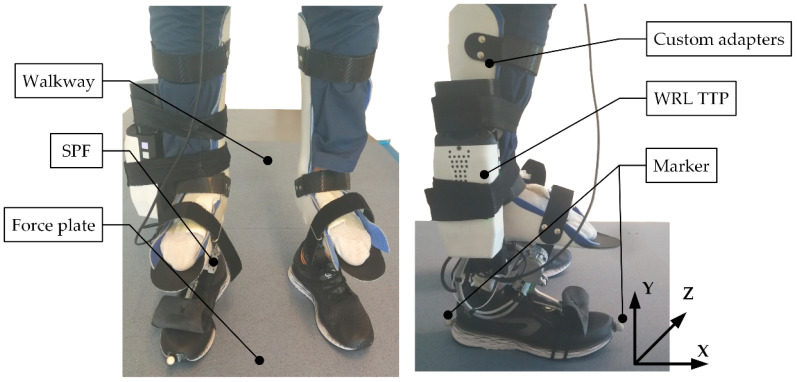
A healthy subject wearing the experimental setup above four force plates: on the right foot, a custom adapter is connected to the WRL TTP and the SPF; on the left foot, the custom adapter is connected to an ESAR foot.

To take part in the experimentation, participants were requested to wear two custom adapters, one for each leg, purposively designed to allow able-bodied subjects to walk with the ankle-foot prosthesis (Figure 6). The adapter was mounted on the WRL TTP on the right leg, featuring an ESAR prosthetic foot instrumented with SPF technology. On the left side, the adapter was mounted on a commercial passive ESAR foot (Pro-Flex XC, Ossür^®^, Reykjavik, Iceland).

It is worth noting that even if the volunteers wore the WRL TTP on the right side, its DC motor was powered off all the time. Therefore, given its patent-pending working principle, the WRL TTP behaved similarly to a commercial Pro-Flex XC foot.

The experimentation protocol included one session for each participant, lasting approximately 2 h. Each participant was first instructed about experimental objectives and procedures, and then they signed an informed consent. Then, participants wore the adapters and devoted up to 15 min to familiarizing and walking with the device. Once the participants felt confident, they were asked to walk back and forth along a 10-m walkway at their preferred comfortable speed about 20 times. The central part of the walkway was instrumented with four force plates (P6000, BTS Bioengineering, Milan, Italy), placed in a 2 × 2 configuration. Volunteers were instructed to step on the array of force platforms at least once for each one of the 20 repetitions. The test was repeated in two different conditions, i.e., with (W S.) and without (W/o S.) wearing the shoes, to assess the potential impact of the shoes on the accuracy of the gait events detection.

### 2.5. Data Analysis

Data analysis was performed offline in MATLAB (MathWorks Inc., Natick, MA, USA). The force platform and motion capture system data were sampled at 200 Hz, whereas the DAB acquired pressure sensors and IMU data were sampled at 100 Hz.

For each of the 20 repetitions, we compared the output of the SPF with one of the force platforms only when the prosthetic foot sole—during the stance phase—was entirely contained within the perimeter of the force plates.

For the force platform, the HS and TO events were detected assuming a threshold of 20 N. Moreover, the trajectories of the heel and tip markers were used to compute the longitudinal axis of the foot, and therefore, to estimate the longitudinal component of the CoP measured using the force platform data (namely, the $CoPy_{FP}$).

For both the force platform and the SPF data, we also computed the stance duration (St) as the time elapsed between HS and TO.

SPF performance was benchmarked against the force platform signals by computing—for each subject—the median absolute error (MAE) and the interquartile range (IQR) of: (i) the time difference in the computation of the HS and TO, and (ii) the St. Then, we grouped data from all subjects by computing the median of all the individual results. The above metrics were computed for both PS- and IMU-based segmentation algorithms. In addition, for the PS-based algorithm, MAE was computed separately for the conditions with and without shoes. Friedman’s test was used to investigate statistically significant differences between the two conditions. Non-parametric statistical descriptors were used because of the rejection of the null hypothesis of normality tests on the distributions of the computed parameters (Lilliefors test, α=0.05).

We also assessed the capability of the SPF to estimate vGRF and CoPy. Before computing vGRF and CoPy, pressure signals were low-pass filtered (2nd-order Butterworth filter, cutoff frequency: 25 Hz) and resampled over 1000 samples between HS and TO. Performance for the evaluation of CoPy was assessed by: (i) computing the Pearson correlation coefficient ρ(CoPy) between the CoPy computed through the force platform ($CoPy_{FP}$) and the pressure sensors ($CoPy_{PS}$) in analogy to [28], and (ii) comparing the root mean square error (RMSE) between the $CoPy_{FP}$ and the $CoPy_{PS}$. Performance for the evaluation of vGRF was assessed by: (i) computing the Pearson correlation coefficient ρ(vGRF) between the vGRF computed through the force platform ($vGRF_{FP}$) and the pressure sensors ($vGRF_{PS}$), and (ii) comparing the peak of the vGRF normalized by the body mass to those grouped across all subjects between the force platform ($vGRF_{FP}^{peak}$) and SPF ($vGRF_{PS}^{peak}$). All the variables involved in the analysis are included in Table 2.

Finally, we estimated the gait velocity of the subjects using the trajectories of the markers, according to the methods described in [44].

## 3. Results

**Figure 7:**
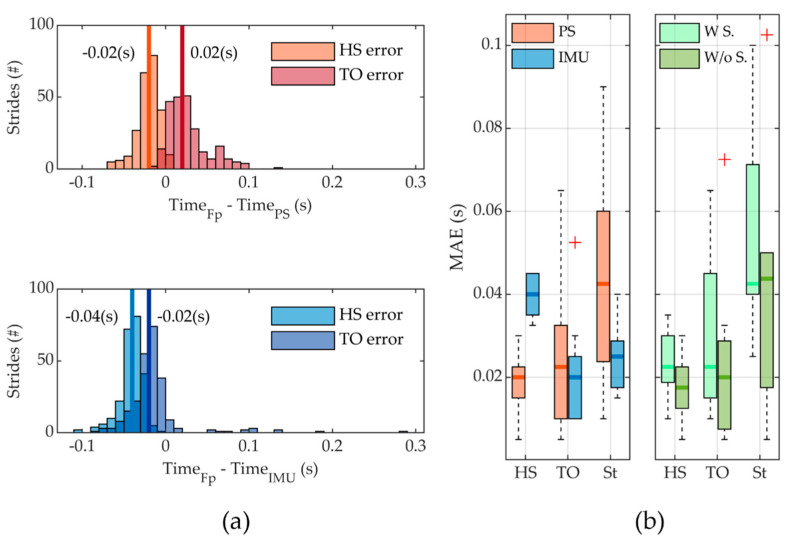
(**a**) Distribution of all time differences of all the strides analyzed, comparing the estimation between the PS (both in W S. and W/o S. conditions) and IMU-based algorithm for the force platform (FP), where a positive time difference means early detection of the event, while a negative time difference is representative of late detection of the event, and median values are in bold; (**b**) boxplots of the median absolute errors (MAE) in the detection of HS, TO, and the stance duration using PS (both in W S. and W/o S. conditions) and IMU, where outliers are reported as red marks. Additionally, MAE distribution is shown for PS separately for the two conditions (W S.–W/o S.).

A total of 244 strides were considered for data analysis for all subjects. Figure 7a reports the error distribution in detecting the occurrence of HS and TO events by both the IMU and the PS-based algorithms. Concerning the detection of the HS, it was detected in delay with respect to the force platform in 100% of the cases for the IMU-based algorithm and in 96.3% of the cases for the PS-based one. The MAE (IQR) was equal to 0.040 (0.010) s (2.45% of the stride period) and 0.020 (0.008) s (1.33% of the stride period) for the IMU- and PS-based algorithms, respectively. The TO events were detected in delay in 89.8% of cases and in advance in the remaining cases for the IMU-based algorithm; MAE resulted equal to 0.020 (0.015) s, corresponding to 1.74% of the stride period. For the pressure sensors-based algorithm, the fraction of late and early detections was 6.6% and 93.4%, respectively, and the MAE was equal to 0.023 (0.023) s, corresponding to 2.05% of the stride period.

Figure 7b reports the boxplots for the MAE and the IQR in the HS, TO, and St computations for all the strides. Regarding the estimate of St, both algorithms of the sensory apparatus resulted in underestimating the actual value. The MAE was equal to 0.025 (0.011) s (corresponding to 1.44% of the stride period) and 0.043 (0.036) s (corresponding to 2.83% of the stride period) for the IMU and PS-based algorithms, respectively. Concerning the PS-based algorithm, we also reported data for the two different walking conditions, namely with and without shoes. Apparently, in the condition without shoes, there is a lower MAE in the identification of HS and TO and the computation of the St. However, this difference was not supported by statistical significance (*p* = 0.53).

**Figure 8:**
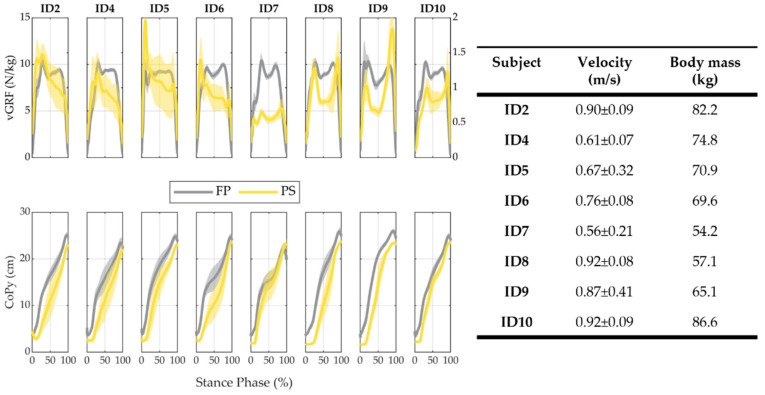
Mean and standard deviation profiles of vGRF (normalized to subject’s weight) and CoPy recorded for each participant (ID). For each ID, mean velocity and standard deviations are shown on the right, as well as their body mass. Yellow and grey data show PS and FP results, respectively. For the vGRF profiles, the *y*-axis on the left of the figure is relative to the FP estimate, while the *y*-axis on the right is for PS estimates. For each ID, all the strides are considered. For the CoPy profiles, the *y*-axis is common for both the FP and PS estimates.

Figure 8 reports the results of the CoPy and vGRF signals, as well as the mean velocity and standard deviation and the body mass for each participant (ID). The vGRF measured with the prosthetic foot was markedly lower than the one measured by the force platform. The median (IQR) value of the peak of vGRF ($vGRF^{peak}$) between all subjects was 1.27 (0.40) N/kg for the PS and 10.37 (0.48) N/kg for the FP. The Pearson correlation coefficients between the vGRF profiles measured by the FP and the SPF are reported in Table 3, together with the $vGRF^{peak}$ detected both from the PS and the FP. The median (IQR) value of the correlation coefficient across all subjects was equal to 0.58 (0.17). Figure 8 also reports the results about the CoPy profiles. Table 4 reports the Pearson correlation coefficient for CoPy computed by FP and SPF for each subject and the RMSE between the FP and SPF profiles; the median (IQR) value of the correlation coefficient across all subjects was equal to 0.97 (0.02), and the RMSE was 4.55 (2.24) cm.

## 4. Discussion

This work presents a novel sensory apparatus—the SPF—for prosthetic feet, integrating pressure sensors and an IMU. Able-bodied participants tested the whole system, and the performance of two gait segmentation algorithms was reported. Hereinafter, we discuss three main aspects of the proposed design: its actual implementation in robotic prostheses, its performance in detecting gait events, and estimating other relevant biomechanical variables such as vGRF and CoPy.

### 4.1. Mechatronics Design

The main objective of this work was the design and development of a sensory apparatus that could be integrated into a variety of ESAR feet for improving the control of lower-limb robotic prostheses. The novelty of the work mainly relies on the combination of two sensing technologies, IMU and pressure sensors, with the perspective of increasing the reliability and robustness in the decoding of gait events during the stance phase of the foot.

The usability of the proposed solution for application in robotic prosthetics is supported by two arguments highlighted by the development process.

The first fact is that the proposed design is modular, and it can be adapted to the specific geometry of a commercial ESAR foot. Indeed, by combining single and 2 × 2 matrices of tactels, we succeeded in instrumenting a Pro-Flex XC (Ossür, Reykjavik, Iceland) with 16 tactels, a number sufficient to cover the most stressed plantar areas of the prosthetic foot [24,35]. The instrumentation of the commercial prosthetic foot with the pressure sensing tactels also required the design and development of a custom foot cover, functional to the housing of the sensing elements under the foot sole. However, the design effort brought a solution whose overall weight and encumbrance are comparable to one of the off-the-shelf prosthetic feet and consequently compatible with a vast majority of commercial shoes, thus overcoming a limitation of other pressure-sensitive in-shoes technologies [24,36].

The second fact is that the SPF appeared to be a good prospect for a reliable foot-sole pressure measurement. Indeed, when approaching the design of the SPF, we had clearly in mind that the overall reliability of an instrumented foot—based on a PCB sole—is much dependent on the durability of the electrical tracks for the application of repetitive mechanical loads, with the latter being caused by the bending of the sole during the stance phase. This led us to opt for a layout of the sensing elements relying on a sum of the individual and matrices of sensing elements. This solution has the advantage of limiting the total path of the electrical tracks, thus reducing the applied mechanical stress [27,28]. In addition, during the construction of the prototype, we also paid attention to route the wires connecting the printed boards to the processing unit in such a way that they were poorly affected by mechanical stress during walking. Although longer experimental sessions are required to assess the overall system reliability, the experiments carried out in this work proved that the proposed design can be viable to achieve adequate reliability for its use in daily life scenarios. Indeed, out of 16,000 steps during the experimentation, no failure of the electrical tracks was recorded. However, it is worth noting that 3 out of 16 sensing elements broke during the experimentation of ID7 (Figure 8); the failure was due to loose welding spots for three electronic components. This issue was unexpected and primarily resulted from a welding process manually executed. After the session with ID7, we repaired the device by replacing the broken wires; no further breakages were found afterward.

### 4.2. Gait Segmentation

Two different sensing modalities were implemented in the presented platform, i.e., pressure sensors and IMU. For each sensing modality, a rule-based segmentation algorithm was developed to detect the events of HS and TO. HS was estimated with a MAE of 0.020 (0.008) s and 0.040 (0.010) s, using PS and IMU-based algorithms, respectively. TO was assessed with a MAE equal to 0.023 (0.023) s for the PS-based algorithm and 0.020 (0.015) s for the IMU-based algorithm. These results are consistent with studies available in literature, which show an error in the identification of HS and TO that is equal to about 0.100 s [26,30,36,37]; this confirms that the sensory system can be employed to control robotic prostheses.

Further analysis of the results shows that the PS-based algorithm detected the HS slightly late and the TO slightly earlier, thus causing an underestimation of the stance duration. This is the consequence of the cascaded application of two thresholds, namely $V_{T}$ and $vGRF_{T}$. Both thresholds are needed to make the reading of PS tactless robust to noise. $V_{T}$ is the threshold value to consider the tactel active; $vGRF_{T}$ is the minimum value of vertical ground reaction force to consider the foot in contact with the ground. The combination of these two thresholds causes a delay in the detection of the onset of the stance phase and earlier detection of the onset of the swing phase. However, the resulting estimated stance time has an error of about 0.043 s, which corresponds to about 2.83% of the gait stride, fully aligned with the performance of other similar technologies [45]. In our study, we also assessed whether wearing a pair of shoes could worsen the performance in the detection of HS and TO, and in the estimate of St. Results have shown that while MAE in the estimate of these three parameters increases when wearing shoes, this change is not statistically significant. Eventually, we compared the achieved performance with the ones of a foot sole based on the same PS technology [28]. Thanks to the fact that in this study the connection between the transducer and the data recording electronics is wired and relies on an SPI link, while in the previous study was wireless and based on an Ultra-Wide-Band link, we succeeded in achieving a smaller MAE in the detection of HS and TO, with a reduction of about 0.040 s and 0.017 s, respectively.

Concerning the performance of the IMU-based algorithm, although our algorithm uses velocity-invariant signal windowing and idles, the results showed a reasonable error, lower than 0.1 s in the detection of the two gait events, as an average between subjects and gait velocities. Velocities resulted in the range 0.5–1 m/s, which is a typical range of self-selected walking speed in lower-limb amputees [41]. The MAE for the detection of HS and TO, and the consequent computation of St, was in the range of 0.040 s, 0.020 s, and 0.025 s, which is compatible with the ones of other state-of-the-art solutions [45].

A major limitation of this study is the lack of an actual merge/fusion of the two sensing modalities into a single classifier, which can possibly bring an increase in the overall system reliability. Although merging the two sensing modalities will be the objective of future investigations, results collected in this study prove that the fusion is feasible. An important prerequisite to fuse sensing modalities is that they can have comparable performance, and this has been proven by the fact that through both pressure sensors and IMU signals we can detect HS and TO with an error that is lower than 0.1 s. The future effort to fuse the two sensing modalities shall develop along two directions. On the one hand, we need a diagnostics tool that can alert when a malfunction is affecting one of the two sensing modalities so that its output signals can be excluded from the computation. On the other hand, we need either a model-based or model-free classifiers, which can fuse the two modalities with the goal to provide the highest accuracy in the gait segmentation. For this latter case, a possible strategy may derive from generalizing the approach presented by Yan et al. in [46] to the specific case of the SPF, where adaptive oscillators may be used to track any of the periodic signals coming from the IMU, while the pressure sensors could be used to detect the HS and smoothly reset the estimated gait phase. This approach would have the advantage of providing a smooth and continuous estimate of the gait phase.

### 4.3. Vertical Ground Reaction Force and Center of Pressure

The control architecture of a powered robotic prosthesis can benefit from a qualitative estimate of the vGRF and CoPy. Indeed, by combining these two variables, it is possible to infer on which stance sub-phase the prosthetic foot is in, and consequently set the actuators motor command. An example is the work of Ambrožic et al. [47], where a relatively simple rule-based classifier mostly relying on thresholds on vGRF and CoPy allowed the control of powered transfemoral prosthesis with a lockable knee and fully powered ankle.

In this study, results about vGRF and CoPy are similar to the ones achieved in the work Martini et al. [28]. The value of the vGRF is strongly underestimated by the SPF if compared to the force platform (1.27 N/Kg vs. 10.37 N/kg). This could be motivated by the combination of different effects, due to the intrinsic characteristics of the system and sensor technology, and to behavioral aspects. Concerning the system characteristics, the amplitude of the estimated $vGRF_{PS}$ depends on the number of pressure-sensitive elements used under the foot sole; hence, when the sensitive elements do not cover the entire plantar area, the $vGRF_{PS}$ results are underestimated. In this work, the number of sensitive elements was chosen as a trade-off between overall system complexity and sensor redundancy; as a result, a relatively-wide plantar area was not sensorized and the $vGRF_{PS}$ was underestimated for all subjects. In addition, for ID7, 3 out of 16 sensors broke during the experiments, therefore resulting in further underestimation of the $vGRF_{PS}$. Furthermore, concerning the sensor characterization curve, it is worth mentioning that the experimental conditions used to obtain the force-to-voltage relationship of the pressure-sensitive elements were significantly different from the conditions of use during walking; indeed, bench tests to characterize the sensors consisted of applying a normal force on the upper surface of the sensor in quasi-static conditions, while in the conditions of use, shear forces and dynamic loads play a fundamental role in the mechanical response of the tactels, and may result in viscoelastic and hysteretic behavior [27,28,40]. Moreover, the presence of the threshold $V_{T}$ on the voltage signal contributes to limiting the amplitude of the vGRF; the relative impact of such an underestimation is higher with lower vGRF profiles, for example in the case of low subjects’ weight or walking velocity (as in ID7). Finally, it is worth mentioning that the differences in the performance of the system observed for the different subjects could be related to differences in subjects’ capability to walk with custom adapters and to maintain a physiological gait pattern.

Even if the vGRF computed by SPF is underestimated, its trend is similar to the one computed through the force platform, in which the median value of the Pearson correlation coefficient is about 0.6. Results vary across subjects and span a correlation coefficient from 0.42 to 0.71. Moreover, the CoPy results highlight a different scenario: signals from SPF and the force platform overlap much more both quantitatively and qualitatively. Indeed, the median value of RMSE is about 25% of the nominal range and the Pearson correlation coefficient across all subjects is always over 0.9. From a practical viewpoint, this latter finding means that the SPF technology can always provide trustable information on whether the load is mostly on the heel or the forefoot, thus allowing discrimination between early and late stance subphases, with the latter being functional to a refined control of any powered lower-limb prosthesis. Transition from early to late stance is indeed paramount to switch from the weight acceptance phase (when a prosthesis needs to dissipate and/or store energy) to the push-off phase (when a prosthesis has to release or inject a new power flow into the gait stride) [48].

### 4.4. Limitations of the Study and Future Works

This study showed three main limitations of the technology, which need to be further examined in future activities.

**Reliability of the mechatronic sensory apparatus.** The sensor failures observed during the experiments with ID7 are not sufficient to draw straightforward conclusions on the durability of the sensors. Such failures highlighted the need for automatic assembly procedures that could ensure higher quality in the welding process. Furthermore, to draw more conclusive considerations on the life cycle of the sensors, dedicated durability tests should be performed with repeatable and structured bench tests (e.g., gait simulators) and using different replicas of SPF [49,50,51].

**Experimental verification with end-users.** While testing the sensory apparatus with healthy subjects, using custom adapters was a necessary preliminary step for the verification of the technology, and tests with the final end-users are necessary to assess the system performance in the conditions of use.

**Experimental verification in various locomotion modes.** The study was limited to the analysis of the system performance in ground-level walking at their self-selected comfortable speed. While this task is surely the most relevant for daily activities, a broader set of locomotion tasks (e.g., stair negotiation, walking on slopes) should be examined to verify the performance of the system in more realistic walking conditions.

## 5. Conclusions

The paper presented a novel sensory apparatus mainly conceived to control lower-limb robotic prostheses. The system consisted of an ESAR foot that integrated pressure sensor matrices and a single IMU on the rear part of the prosthetic foot. The pressure-sensitive elements are modular and have been integrated between an ESAR prosthetic foot and its cosmetic cover to minimize encumbrance while maintaining the usability of the standard passive foot. Two dedicated threshold-based algorithms have been developed based on the pressure sensors and the IMU signals to detect relevant gait events: heel strike (HS) and toe-off (TO). The system was tested with eight healthy participants who wore mechanical adapters to walk with prosthetic feet. Results were comparable to other sensing technologies, and they allowed us to recognize HS and TO with a time error in the range of tens of ms [26]. The proposed technology also allowed us to estimate the relevant biomechanical variables such as vGRF and CoPy with a level of accuracy that is sufficient to properly control a powered robotic prosthesis. Future works shall focus on a more extensive experimentation of the technology along three directions. First, we need to provide longer tests, including bench tests, to assess the system reliability over a much higher number of steps. Second, in order to enhance the overall system reliability, it will be important to develop and validate an algorithm fusing together data from pressure sensors and IMU. Finally, the system shall be experimented with amputees and its output employed to actually control a powered lower-limb prosthesis.

## Figures and Tables

**Table 1:** List of parameters implemented into the IMU-based algorithm.

Parameters	Description	Unit
$a_{x}$	Acceleration along IMU *x*-axis	m/s^2^
$a_{y}$	Acceleration along IMU *y*-axis	m/s^2^
$a_{z}$	Acceleration along IMU *z*-axis	m/s^2^
$a_{T}$	Acceleration threshold	m/s^2^
$\omega_{x}$	Angular velocity along IMU *x*-axis	deg/s
$\omega_{xT\;MSw}$	Mid-swing angular velocity threshold	deg/s
$\omega_{xT\;TO}$	Toe-off angular velocity threshold	deg/s

**Table 2:** Summary of the variables and parameters used in the analysis.

Parameters	Description	Unit
ρ(vGRF)	Pearson correlation coefficient for vGRF	-
ρ(CoPy)	Pearson correlation coefficient for CoPy	-
$CoPy_{FP}$	CoPy estimated by the force platform	cm
$CoPy_{PS}$	CoPy estimated by the pressure sensors	cm
$vGRF_{FP}$	vGRF estimated by the force platform	N
$vGRF_{PS}$	vGRF estimated by the pressure sensors	N
$vGRF_{FP}^{peak}$	Peak value of vGRF estimated by the force platform	N
$vGRF_{PS}^{peak}$	Peak value of vGRF estimated by the pressure sensors	N

**Table 3:** Median (IQR) of the correlation coefficients between the vGRF estimated by the pressure sensors and the one recorded from the force platform, ρ ($vGRF_{FP}$, $vGRF_{PS}$ ), and peak vGRF for both pressure sensors and the force platform ($vGRF_{PS}^{peak}$, $vGRF_{FP}^{peak}$ ), for each subject (ID).

Subject (ID)	ρ $(vGRF_{FP}$ $,\;vGRF_{PS})\;[0,\;1]$	$vGRF_{PS}^{peak}\;(N/\mathrm{kg})$	$vGRF_{FP}^{peak}\;(N/\mathrm{kg})$
ID2	0.71 (0.32)	1.39 (0.28)	10.63 (0.71)
ID4	0.65 (0.23)	1.28 (0.56)	10.36 (0.77)
ID5	0.45 (0.50)	1.90 (0.47)	10.19 (1.06)
ID6	0.54 (0.21)	1.60 (0.56)	9.77 (0.44)
ID7	0.58 (0.30)	0.66 (0.10)	10.65 (1.01)
ID8	0.58 (0.19)	1.26 (0.21)	10.14 (0.83)
ID9	0.42 (0.14)	1.11 (0.23)	11.45 (1.80)
ID10	0.68 (0.18)	1.08 (0.25)	10.37 (0.31)
Overall	0.58 (0.17)	1.27 (0.40)	10.37 (0.48)

**Table 4:** Median (IQR) of the correlation coefficients and RMSE between the CoPy estimated by the pressure sensors ($CoPy_{PS}$ and the one recorded from the force platform $CoPy_{FP}$ for each subject involved in the experimentation (ID).

Subject (ID)	ρ $(CoPy_{FP}$ $,\;CoPy_{PS})\;[0,\;1]$	$\mathrm{RMSE}\;(CoPy_{FP}$ $,\;CoPy_{PS})\;(\mathrm{cm})$
ID2	0.97 (0.06)	4.77 (2.77)
ID4	0.96 (0.03)	4.33 (1.62)
ID5	0.97 (0.02)	4.11 (1.09)
ID6	0.94 (0.03)	5.63 (1.75)
ID7	0.98 (0.01)	2.40 (1.09)
ID8	0.96 (0.02)	5.66 (1.97)
ID9	0.96 (0.03)	5.46 (2.23)
ID10	0.99 (0.01)	2.51 (1.46)
Overall	0.97 (0.02)	4.55 (2.24)

## Data Availability

The datasets generated during and/or analyzed during the current study are available from the corresponding author on reasonable request.
